# A Survey of Data Semantization in Internet of Things

**DOI:** 10.3390/s18010313

**Published:** 2018-01-22

**Authors:** Feifei Shi, Qingjuan Li, Tao Zhu, Huansheng Ning

**Affiliations:** 1 School of Computer and Communication Engineering, University of Science and Technology Beijing, Beijing 100083, China; shifeifeiustb@163.com (F.S.); b20150313@xs.ustb.edu.cn (Q.L.); 2 Beijing Engineering Research Center for Cyberspace Data Analysis and Applications, Beijing 100083, China; 3 Software School,University of South China, Hengyang 421001, China; tzhu@ustb.edu.cn

**Keywords:** Internet of Things, data semantization, ontologies

## Abstract

With the development of Internet of Things (IoT), more and more sensors, actuators and mobile devices have been deployed into our daily lives. The result is that tremendous data are produced and it is urgent to dig out hidden information behind these volumous data. However, IoT data generated by multi-modal sensors or devices show great differences in formats, domains and types, which poses challenges for machines to process and understand. Therefore, adding semantics to Internet of Things becomes an overwhelming tendency. This paper provides a systematic review of data semantization in IoT, including its backgrounds, processing flows, prevalent techniques, applications, existing challenges and open issues. It surveys development status of adding semantics to IoT data, mainly referring to sensor data and points out current issues and challenges that are worth further study.

## 1. Introduction

Internet of things (IoT) is bringing Internet truly into our routine life by deploying intelligent equipments ranging from multi-modal sensors to white intelligent goods [1]. As Cisco predicts in [2], over 50 billion devices will be joined into the Internet before 2020. Five hundred zettabytes of data will be produced by tremendous machines, devices, and even the interactions between them. Moreover, the developments of IoT give birth to intelligent realms such as smart transportation [3], e-health [4] and smart homes [5] which are aimed at providing users with better service and higher quality of life.

However, due to the lack of interoperability, information generated by different sensors or devices cannot be shared with each other, which has become a severe challenge nowadays. Although nearly 45% data created on the Internet can be processed, it is tough work to mine and dig out the hidden information behind them. Moreover, cross-domain knowledge becomes increasingly difficult to share with others because of the heterogeneity of data.

To achieve a better interpretation of heterogenous data, more and more researches start to focus on techniques enabling machines to intelligently understand IoT data. Among all approaches, adding semantics to IoT data is one of the most prevalent methods. Known as an extension of the World Wide Web [6], the semantic web resolves isolation problems between heterogenous information and provides a better understanding of surroundings. By adding general mark-ups and notifications, semantization makes it possible for machines to understand and interpret heterogenous data and prompts cross-domain interactions to a large extent.

This paper illustrates an overview of IoT data semantization, including related concepts, architectures, key techniques, applications and challenges. The main contributions of this survey are as follows:

- It provides a detailed overview of data semantization such as the related concepts and existing architectures for adding semantics to IoT data and summarizes a general processing architecture for data semantization.
- It presents key techniques involved in data semantization including techniques in data collection, data preprocessing and semantic annotation.
- It analyzes challenges and open issues that are worth studying in future work such as standardization and generalization, complexity and dynamicity, and security and privacy.

The remainder of this survey is arranged as follows. Section 2 gives an overview of semantics which consists of the definition, the significance and the general architectures. Section 3 mainly focuses on key techniques involved in data semantization, including technologies in data collection, data preprocessing and semantic annotation. Section 4 lists Smart Homes, E-health and Smart Cities as representative applications of data semantization. Section 5 presents existing challenges and issues that are worthy of researching in future work. Finally, Section 6 concludes the survey.

## 2. Overview

### 2.1. The Definition of Data Semantization

As Berners-Lee once said, “developments will usher in significant new functionality as machines become much better able to understand and process the data” [7], semantics provide potentiality and possibility for machines to mine and dig out hidden information. Data Semantization refers to formatting data with reasonable mark-ups and special properties such as tags, labels and many more. It merges domain knowledge and context information with sensor data, making it easier for machines to understand and process. Moreover, semantics provide a unified description architecture which prompts information and knowledge interaction between variable sensor nodes. Data semantization is regarded as a kind of knowledge organization [8] which is aimed at representing semantic relationships in order to support interoperability between heterogenous data sources.

### 2.2. The Significance of Data Semantization

Data semantization overcomes the barriers brought by data heterogeneity, and it provides possibility for better understanding of ambient environments. With a growing number of sensors and devices connected to the Internet, semantics play more and more essential roles in terms of data integration, data interoperability and data understanding.

- Data Integration
  Data are sensed and gathered from a stakeholder, no matter it is a sensor, a device or triggered by a inhabitant. Therefore, it is vital to seamlessly integrate data and information to a consistent description format [9]. Adding semantics supports data integration by allowing data interoperability between different sources and prompts domain-across applications [10] largely.
- Data Interoperability
  Data interoperability mainly refers to data from different sources being understood and interpreted unambiguously. Since it is demanding to explore implicit meanings of an independent area, information from different domains need to communicate and interact with each other. By adding unified data descriptions, it is possible for different domains [11] such as weather forecasting and healthcare to exchange and share information.
- Data Understanding
  Data semantization means formatting data with fixed mark-ups, thus providing a unified description for sensor data. With semantic notifications, most information can be expressed with a formal specification language, therefore it improves the possibility of data understanding to a great degree. Data semantization facilitates the progress for machines to accept and understand information totally.

### 2.3. General Architecture for Data Semantization

**Figure 1:**
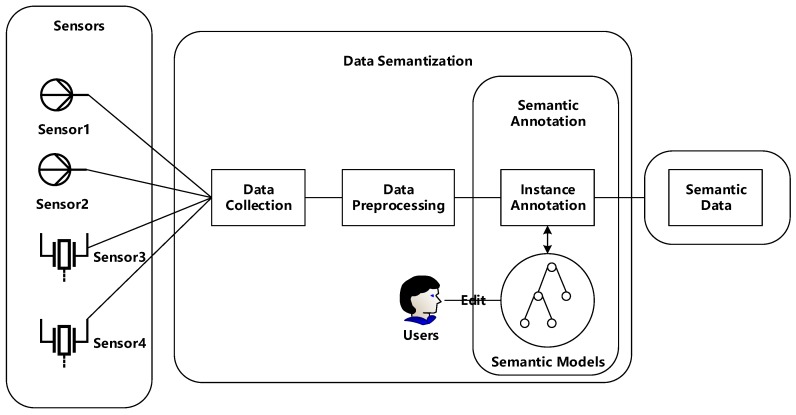
The System Architecture for Adding Semantics.

With the popularity of semantic sensor web, researchers are dedicated to developing a system architecture which could automatically transform sensor data to semantic expression formats. Generally, sensor data are full of diversity, ubiquity and volatility, which poses challenges for machines to understand and process. Data semantization allows users to reason about human’s activities related to sensor events and make effective responses to dynamic environments. More and more studies are concentrating on applying data semantization in smart homes, healthcare and weather forecasting for monitoring inhabitants’ and environments’ abnormity and providing significant guidance and help. Zhang [12] proposes a system architecture used for transforming sensor data to semantic expression formats, which consists of three parts, semantic annotation, producing mapping file and transforming with semantics. Razzak [13] also depicts an architecture dealing with sensor data in which the responsible component for adding semantics is a publisher creating channels to equip data with semantic notifications. Chen [5] presents a conceptual architecture with adding a semantic layer which is responsible for adding semantics to sensor data. Based on current research, we provide a general processing architecture for adding semantics as shown in Figure 1.

As depicted in Figure 1, the processing architecture is composed of three parts. Part 1 is a physical layer, which consists of various sensors responsible for sensing data from ambient environments. Part 2 is the core component named data semantization, which is composed of three steps including data collection, data preprocessing and semantic annotation. The first two steps are regarded as preliminary work of data semantization. Part 3 is the final semantic expression formats after adding semantics to sensor data. Here follows a brief introduction to Part 2, data semantization.

- Data Collection. In this stage, the main work is to sense and gather heterogenous data from diverse sensors including sensor id, value, measurement and other information. For separate sensor nodes, there is no doubt to transfer data to a processer via wired/wireless communication technologies. However, for sensor networks, a main challenge is how to arrange the roles of all sensor nodes based on the requirements and limited resources constraints, as well as the protocols used for communications between networks.
- Data Preprocessing. Data collected from environments are full of uncertainty and noise, which may result in severe problems with regard to data utilization. For example, in applications where data are adopted for trend predictions, the more accurate the data are, the more reasonable the trend is predicted. It is undeniable that anomalies or outliers are essential in the case of discovering abnormal situations, for instance, when the patient’s heart beat is different from normal values, an alarm would inform the doctor. However, there do exist situations where noise, anomalies and outliers need to be tackled and cleaned. By adopting data preprocessing algorithms, accuracy of sensor data would be improved and it is beneficial for further processes.
- Semantic Annotation. Semantic annotation is regarded as the key step in the whole processing architecture, which means adding semantic notifications to preprocessed data. Generally, semantic annotation is composed of two steps, semantic modeling and instance annotation. Semantic modeling serves as an important role, and users may define new or reuse existing semantic models depending on situations. The preprocessed data would be instantiated based on predefined semantic models to finish the process of semantic annotation.

A primary goal of adding semantics to sensor data, especially in Smart Homes and Ambient Intelligence areas, is to create a context-aware environment and help users better understand their surroundings [9]. In order to get deeper insights into data semantization, we give a detailed description of key techniques involved in data semantization in Section 3.

## 3. Key Techniques

As shown in the general architecture, the process of data semantization includes three parts, namely data collection, data preprocessing and semantic annotation. In this section, key techniques involved in each part are discussed.

### 3.1. Data Collection

Data collection is the preliminary work of data semantization. It collects and gathers data sensed from ambient environments or objects. In some small scenarios, separate sensor nodes are popular with its convenience and portability. Under such circumstances, data are collected through wired cables or wireless communication modes such as BlueTooth or WIFI. However, separate sensor nodes fail to sense and collect data in large scale applications due to limited capability. With the increasing network scale and practical requirements, wireless sensor networks (WSNs) [14] emerges at the right time aimed at satisfying these requirements.

WSNs are composed of small, low-powered and cheap sensors with limited memory and computing capabilities. Thus, when it comes to data collection techniques in WSNs, energy efficiency needs to be considered. This section mainly focuses on techniques in and between WSNs.

#### 3.1.1. Techniques in WSNs

In WSNs, collecting data from nodes deployed in dispersed areas is crucial. Many researchers pay attention to developing energy-aware communication techniques for WSNs in order to prolong the life of networks. This section presents a brief overview of techniques involved with data collection, mainly referring to the data aggregation protocols in WSNs.

In 2000, Heinzelman [15] proposed The Low-Energy Adaptive Clustering Hierarchy (LEACH) which is a clustering-based protocol. For even distribution of energy among all sensor nodes in WSNs, it adopts a randomized rotation of local base stations, which means that a sensor node randomly decides whether to be head of cluster or not. Other nodes transmit data to the cluster head and only the cluster head performs computation. Compared with traditional communication protocols such as direct transmission and multi-hop routing, LEACH achieves great reduction in energy consumption. However, LEACH shows weakness for time-critical communications.

In 2001, Manjeshwar [16] presented The Threshold sensitive Energy Efficient sensor Network protocol (TEEN) which is well-suited for reactive networks. TEEN is known for enabling nodes to sense data continuously and it only transmits data when the value exceeds hard threshold or the variance between new and old values exceeds soft threshold. Therefore, TEEN saves more energy consumption compared with LEACH. Lindsey [17] also puts forward a Power-Efficient Gathering in Sensor Information Systems (PEGASIS) protocol which is regarded as an improvement of LEACH. In this schema, nodes only communicate with their close neighbor nodes in order to save communication energy.

In 2004, Lu [18] proposed a Dynamic Medium Access Control (DMAC) protocol which overcomes the significant sleep delay in traditional MAC protocols. It designs the sleep schedule based on the depth of data gathering trees. Ye [19] puts forward an Energy Efficient Clustering Scheme (EECS) protocol, which is a novel clustering scheme electing cluster heads according to local radio communication. Experiments shows EECS outperforms LEACH a lot especially when processing periodical data. Qing [20] also chooses clustering algorithms as the main protocol in WSNs and improves it by electing cluster heads based on the ratio among dump energy of each node. This novel protocol is named as distributed energy-efficient clustering scheme (DEEC). Bouldin [21] raises a protocol for distributed sensor nodes named Rank-Based Data Gathering (RBDG). In RBDG, sensor nodes would be given a random rank between 0 and 1. The node getting the highest rank would be selected as the communication node and others are leaf nodes. Bajaber [22] focuses on a clustering protocol named as Efficient Cluster-Based Communication Protocol (ECOMP). In ECOMP, a bidirectional ring is established which enables each node to transmit data to their neighbours, thus the cluster heads do not need to receive data from all its member nodes. Simulations show ECOMP has a great energy consumption reduction compared with other clustering protocols. Different from protocols mentioned above, Chhabra [23] combines cluster and tree based protocols, and it prolongs the lifetime of WSNs by protecting the parent node death slowly.

RajeswariData [24] lists most techniques related to data collection in WSNs and points out current big challenges such as the limited bandwidth and resources, latency and scalability, and so forth. PrasanthA [25] describes tree-based, cluster-based, multi-path and hybrid techniques in data collection and also makes a comparison between their pros and cons.

#### 3.1.2. Techniques between WSNs

No matter which algorithms are chosen in WSNs, communications between different networks are necessary and inevitable. This section focuses on popular message protocols used in IoT enabling transmitting data via networks.

Message Queuing Telemetry Transport (MQTT) [26] is a messaging protocol designed by IBM, which is suitable for devices with limited capabilities. It is based on publish and subscribe model, and supports more than one clients to establish connections with topics of interests. MQTT performs better especially in low-bandwidth and unreliable networks, thus it has wide application space in WSNs. IBM developed a Merlin system which is applied in homecare with sensors. This system transmits data sensed from patients to doctors by MQTT protocol.

The Constrained Application Protocol (CoAP) [27] ia another lightweight protocol for constrained networks with limited resources. The core part of CoAP protocol is UDP which is used to retransmit missing packets. CoAP consists of two layers, Transaction Layer and Request/Response layer. The Transaction layer is responsible for handling single message exchange while the Request/Response layer manage request/response transmission and resource. In [28], it compares the performance of MQTT and CoAP for smartphone-based sensing in terms of bandwidth usage, reliability and round trip time. The experiments show CoAP outperforms MQTT in bandwidth usage and round trip time while MQTT performs better in reliability.

Besides communication protocols mentioned above, other protocols such as RabbitMQ [29] are also popular. RabbitMQ is a lightweight protocol which adopts the standard of Advanced Message Queuing Protocol (AMQP) [30]. It is known for advantages of supporting distributed deployment and asynchronous messaging. In [31], simulations reveal that RabbitMQ has a higher performance in producing messages, and is appropriate for collecting data in WSNs. With the variety of existing WSNs protocols, energy consumption, constrained resources and network features should be considered when choosing the most suitable one.

### 3.2. Data Preprocessing

Techniques in adding semantics to sensor data depend on data sets which are supposed to be complete and clean. However, the environments are full of noise and uncertainty and data collected are imprecise, uncertain and incomplete. Therefore, data preprocessing seems essential before data semantization. In [32], authors propose an architecture of data preprocessing including dimensionality reduction, feature extraction and so forth. This section concentrates on preprocessing techniques dealing with noisy and incomplete data, as well as approaches to data dimensionality reduction.

#### 3.2.1. Noisy Data Cleaning

Sensor data are full of inaccuracy which may arise from the inherent defects of sensors, the failures of network links and the limits of discharged batteries and so on. In addition, fluctuations of the environments would have an influence on the precision of observed measurements. As the source of sensor networks, the accuracy of sensor data has dramatic effects on the performance of following operations. In WSNs, outliers are defined as values which are quite different from other values. Although in some cases, outliers are important for detecting abnormal situations, there exist applications which have high requirements for the accuracy of data. Under such circumstances, anomalies and outliers are regarded as noise which need to be cleaned. A straightforward approach to noisy data cleaning is describing an area which covers all normal situations. Therefore, a new situation that does not belong to the predefined range can be classified as noisy data. However, it is almost impossible to encompass all possibilities of normal situations. The boundary between normal and abnormal data is not distinct. Thus, a model-based approach emerges. In the model-based data preprocessing approach [33], there exists a well-established model which is used to infer most probable sensor values. By comparing real sensor data and inferred values, anomalies can be found and located. This section mainly surveys two types of models, regression models and probabilistic models, which are representatives in sensor data cleaning.

Regression Models:

Usually, sensor values have dependency on other factors such as time, position and other sensor data. In regression models with already existing samples, a relationship model is computed as a predefined standard for sensor values prediction. When it comes to regression models, the most popular functions are Polynomial Regression and Chebyshev Regression. This section mainly focuses on Polynomial Regression. Polynomial Regression is known as a form of analysis describing the regression relationship between independent variables *x* and *y*, and the relationship is depicted as an *n*th degree polynomial in *x*. The general expression can be shown as follows:

(1) $$y=\beta_{0}+\beta_{1}x+\beta_{2}x^{2}+,...,+\beta_{n}x^{n}+\epsilon$$

Moving average (MA) is a method of low-pass filtering, calculating average of already known data sets based on Polynomial Regression theory. By drawing a mean line of a series of sensor values, it is possible to predict sensor values and find outliers. However, the general MA algorithm fails to satisfy the requirements of high efficiency and instant response in sensor-related areas. In [34] Zhuang proposes a Weighted Moving Average-based approach. The Weighted Moving Average (WMA) with confidence is an improvement of traditional MA algorithm. The main idea of WMA is to locate important sensor values and give them a higher weight, thus the important sensor values would be reflected as quickly as possible. Experiments show that compared with original MA, WMA uses fewer samples and takes up less time to make instant response.

In addition, Pumpichet [35] presents a novel data cleaning method based on the Normalized Least Mean Square (NLMS) linear regression model. In this technique, a virtual static sensor is designed to cooperate with a predication model. When a data stream comes, the base station starts to clean missing data if any are detected. It finally would choose a most suitable virtual sensor to do data cleaning based on the predefined predication models.

Probabilistic Models:

Regression models mentioned above provide concrete sensor values used as standards to detect whether there exist anomalies. However it may regard some normal sensor values as anomalies mistakenly. For enhancing accuracy in detecting noisy data, many researches choose probabilistic models instead of regression models. Probabilistic models compute a probability distribution, and if the coming sensor value resides in the accurate area it would be accepted. In [33], the error bound is defined as 3σ. If the coming sensor value falls outside the error bound, it is regarded as an outlier. Among all probabilistic models, Kalman filter [36] is a popular algorithm used for outlier detection. Kalman filter assumes that the true value at time *t* has dependency with the state of time t-1, and the model is illustrated with parameters $F_{t}$, $B_{t}$ and $w_{t}$.

(2) $$x_{t}=F_{t}x_{t-1}+B_{t}u_{t}+w_{t}$$

where $F_{t}$ is the state transition metric applied to time t-1, $B_{t}$ is control-input model and $w_{t}$ is the process error which satisfies Gaussian Distribution. Lin [37] implements an initial toolkit with Kalman filter and regression models, and conducts experiments based on temperature, humidity, light and other sensor values. The experiments prove Kalman filter has promising performance compared with regression models. In addition, Aggarwal [38] gives a conclusion of probabilistic models including Probabilistic Mixture Modeling for Outlier Analysis, Mahalanobis method and expectation-maximization algorithm in terms of application scenarios and strengths. Although probabilistic models reduce the fault positives, the accuracy and precision still need to be improved.

#### 3.2.2. Missing Data Completing

Apart from noisy data, the presence of missing data is very common in real applications, which is also named as incomplete data [39]. There are various reasons for missing data, including sampling errors, network failures or device faults. It is impossible to avoid missing data phenomenon while the difficulties to tackle it are immense. Wrong process for incomplete data may result in poor knowledge and unreasonable conclusions [40]. Nowadays more and more researches focus on developing techniques dealing with incomplete data.

The first option is to ignore missing data, however this technique shows little benefits because discarding data values may produce bias in subsequent process [41]. It would eliminate useful information which is recorded in missing data. Another similar technique is labeling missing data with “Unknown”. It seems to have the same influence with ignoring missing data since they provide no useful information to help the interpretation of incomplete data.

A more popular technique of preprocessing incomplete data is to estimate the missing data value. Usually, statistical approaches are used in predicting missing values [42]. It calculates the probability function based on existing data and makes a prediction on the missing data. Jonathan [43] proposes a novel approach dealing with incomplete data with Bayesian and maximum likelihood parameter estimation which adopts data augmentation into the covariance matrix and gives likelihood-based inference for incomplete data. To sum up, noisy and incomplete data are all imperfect data that need to be paid high attention. Research in dealing with imperfect data still has a long way to go.

#### 3.2.3. Data Dimensionality Reduction

With the increasing size of data sets, high dimensionality of data has severe impacts on subsequent processing [44]. It would influence the efficiency of processing algorithms by adding computational loads. Therefore necessary dimensionality reduction is useful when dealing with large data sets. Here, we focus on two popular approaches, that is Feature Selection (FS) [45] and Space Transformation (ST) [39].

FS is regarded as variable selection in machine learning or statistic methods. By selecting several representative features, it achieves the simplification of models and reduces the training complexity. More important, reduction of data dimensionality helps a lot in avoiding the phenomenon of overfitting. FS helps remove irrelevant or redundant features thus it reduces the energy consumption in later process. Liu [46] concludes categories of FS algorithms into two types, supervised algorithms and unsupervised algorithms. In [47], an overview of existing FS algorithms such as filter, wrapper and embedded methods is described. By selecting important features, FS algorithms make models much easier to be processed, interpreted and understood.

Apart from FS, ST is another way to reduce data dimensionality. The main idea of ST is to generate new features from original ones. Generally ST algorithms are composed of linear methods and nonlinear ones. Linear methods are mentioned in [39] including factor analysis [48] and Principal Components Analysis (PCA) [49]. Some researches concentrate on nonlinear ST algorithms such as Locally Linear Embedding (LLE) [50]. All approaches related to ST are changing original feature sets to a smaller one in order to reduce data dimensionality, with considerations of geometrical properties or other information.

### 3.3. Semantic Annotation

Semantic annotation overcomes many of the barriers created by heterogenous sensor data. With the widespread of sensors deployed in areas like smart homes and smart cities, translating sensor data using semantic notifications is becoming an overwhelming tendency. Researchers are beginning to focus more efforts on developing and improving technologies in adding semantics. Usually, semantic annotation is composed of two steps, creating semantic models and ontology instantiation. This section gives a brief introduction on semantic expression formats of sensor data, and presents representative semantic models in terms of activities and context.

#### 3.3.1. Semantic Expression Formats

Resource Description Framework (RDF) [51] is a language used to give descriptions of web resources. It is the most widely used data model for representing semantic sensor data with the format of triples, which consist of subject, property and object [1]. To be brief, the format of triples conveys a relationship that the subject has a property whose value is the object, which is also called a measurement. For instance, “Sensor 1” is a subject with a property of “humid”, and its value is “40.1”. There are several different syntaxes [52] used for writing and serializing RDF data. RDF/XML [53] is the oldest syntax representation, which encodes RDF and existing XML elements together and it is the most widely used syntax nowadays. As the simplest way to represent context information for IoT devices or sensors, it is easily to be understood and processed by machines. Thus, many experiments set up at Smart Homes and intelligent environments adopt these expression formats. Zhang [54] mainly focuses on transforming sensor data to RDF in his experiments. Satterfield [55] designs a smart home system in which it models sensor data to RDF triples. RDF only concentrates on relationships of triple predicates, whereas RDF Schema (RDFs) [56] is described as an extension of RDF which focuses on illustrating subclass hierarchy and attribute hierarchy, as well as the definition of domain and range. RDFs is more suitable in elaborating relationships like subclass, subproperty and so forth.

Sensor Makeup Language (SenML) [57] is another expression format designed for simple sensor measurements, whose advantage is to achieve a balance between useful and auxiliary information carried by sensors. Thus this language model is preferred by processors with limited capabilities. Similar with RDF, it also has different representation formats such as Javascript Object Notation (JSON) [53], eXtensible Markup Language (XML), and Efficient XML Interchange (EXI) [58].

Apart from data formats mentioned above, Entity Notation (EN) [59] is also a technique which is applicable for resource-constrained sensors. Compared with other alternatives, EN has two representation formats, complete packets and short packets. Complete packets are mainly responsible for connecting with higher level ontologies, thus they need to provide detailed descriptions and information, while, on the other hand, short packets focus more on crucial items such as identifiers, variables and templates, which are predefined for the conversion from short packets to complete ones.

With the increasing demand for better expressivity, the data formats mentioned above show limited capabilities in the case of complicated knowledge modeling, therefore the Web Ontology Language (OWL) [60] emerges at the right time to handle this issue. It allows doing logic inference based on predefined reasoning rules, which makes it possible to acquire implicit knowledge. As a standard of ontology modeling, OWL aims at achieving a balance between expressivity and scalability. It is composed of three language subsets, OWL FULL, OWL DL and OWL Lite. As shown in Table 1, OWL FULL has the strongest expressivity, with no mandatory type separation and none restrictions on items usage. While OWL Lite has the most restrictions on items usage and mandatory type separation, it has the weakest expressivity. On the contrary, OWL Lite has the strongest reasoning ability, while OWL FULL has the weakest ability of inference among these three sub languages.

**Figure 2:**
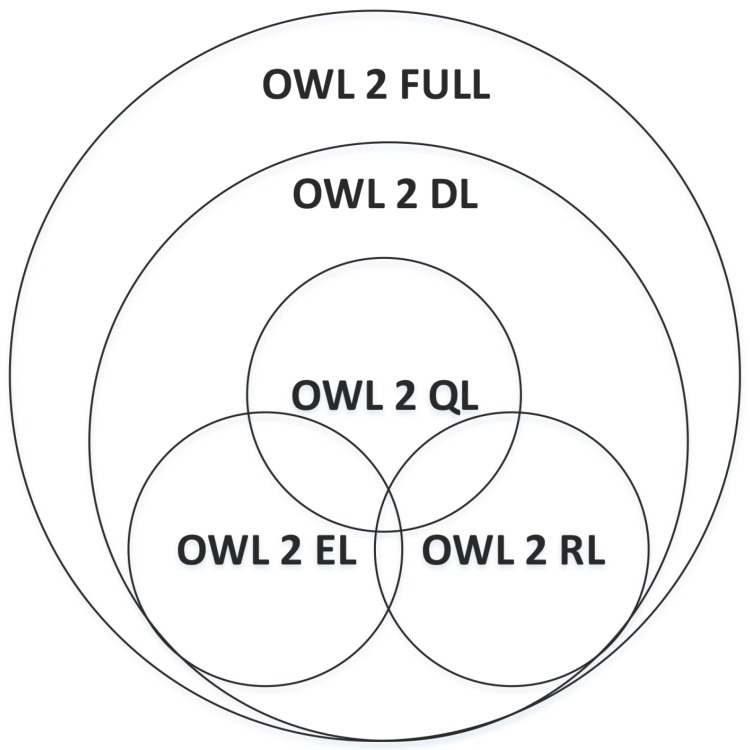
Relationships between sub-languages of OWL 2.

In 2012, W3C published a new version of OWL named OWL 2. Due to the limited expressivity of OWL Lite, OWL 2 mainly consists of OWL 2 FULL and OWL 2 DL. The main difference between them is the way to express semantics of ontologies. OWL 2 FULL adopts the RDF-Based Semantics whereas OWL 2 DL takes the Direct Semantics. OWL 2 DL is composed of three sub languages, OWL 2 EL, OWL 2 QL and OWL 2 RL in order to improve the computation capability. The relationship between them is shown in Figure 2.

As semantic data formats need to be applied in the real world, several evaluation criteria have been given which provide guidance for choosing the most suitable semantic expression format, namely whether it can be transformed into conceptual graphics, whether it can express semantics, as well as the expressivity and energy consumption. Su [1] compared RDF, SenML and EN on overall energy consumption, and demonstrated EN, with short packets, consumes the least energy, whereas SenML with EXI ranks first in energy consumption. Compared with RDF, SenML, EN and OWL, OWL 2 has a stronger ability to express more complicated models, thus it needs more energy.

#### 3.3.2. Semantic Models

Semantic modeling is recognized as the core part of semantic technology and semantic sensor web [33]. Among various models, ontologies are the most representative ones. An ontology is a specification of a conceptualization [61], which is regarded as a mechanism for knowledge sharing and information interaction. Ontology modeling has become a pervasive approach for data semantization in areas such as health care, smart homes and ambient intelligence. This section gives a detailed introduction of representative ontologies, as well as automatic ontology editors.

(1) Representative Ontologies:

As semantic modeling is becoming more and more popular in the filed of ambient intelligence, we give an illustration on representative ontologies assisting in activity recognition and situation-awareness.

- Ontologies for Activities
  In this part, user-centric ontologies are introduced which mainly focus on users. It is known that activities are triggered by users with different operation sequences and manners. To improve the activity ontologies, it is required to consider influencing factors, such as user profiles, user privacy and so forth. Nowadays, an increasing number of ontologies are designed to help recognize users’ activities in daily life.
  The Standard Ontology for Ubiquitous and Pervasive Applications (SOUPA) [62] represents intelligent agents with associated beliefs, desires, and intentions, as well as time, space, events, user profiles, actions, and policies for security and privacy. One advantage of SOUPA is that it supports combination with pervasive environments. CoBrA-Ont [63] is an extension of SOUPA which defines key categories like agent, action, device, time, space, and so forth. The distinct improvement of CoBrA-Ont is it integrates considerations of users’ privacy by restricting the sharing of information sensed by hidden sensors or devices. Preuveneers [64] also proposes an ontology named CoDAMoS targeted at the description of four components, user, environment, service and platform. The main advantage of this ontology model is it describes two levels of granularity, tasks and activities. Another ontology put forward by Lewis [65]-The Delivery Context Ontology-provides a definition of device characters, environment, hardware and so on. In 2011, Riboni [66] proposed an ontology for human activity recognition named PalSPOT ontology. It involves descriptions of individual and social activities such as comment, proposal or request for information. However, all ontologies mentioned above ignore the situation with incomplete knowledge, thus Rodríguez [67] establishes a fuzzy model that enables modeling uncertain and vague knowledge. In 2014, Natalia [68] made a comparison between important ontologies in terms of the components and items modeled in them.
- Ontologies for Context and Situation
  Apart from activity models, context and situation ontologies become more and more crucial in expressing semantics. With the awareness of context, it is possible to understand current situations and make instant reactions. In [64] environment concepts such as time, location and environmental conditions are described. Chen et al. [5] presents an ontology including modelings of physical environment, inhabitants, sensors, devices and middleware services. In 2015, a Smart Appliances REFerence (Appliances REFerence) ontology [69] was published with descriptions of smart devices such as meters, switches and other energy controllers.
  Sensors are also objects that need to be semantically described. To provide a standardized expression, the W3C Semantic Sensor Network Incubator Group puts forward the most foundational ontology for sensors named the Semantic Sensor Network (SSN) Ontology [70]. It provides descriptions of concepts such as deployment, device and data. The core pattern in SSN is called the Stimulus Sensor Observation (SSO). In 2017, W3C published a new version of SSN ontology based on Sensor, Observation, Sample, and Actuator (SOSA) ontology [71] and the latest SSN ontology represents actuation models. However, as pointed out in [72], the SSN ontology is lack of descriptions of other fileds of IoT. The IoT ontology [73] expands from SSN, with descriptions of concepts like Physical Entity and Smart Network in order to support semantic expressions for interconnected, aligned and clustered entities. IoT-Lite Ontology [74] is also regarded as an expansion of SSN ontology. In addition to the definition of “ssn:Device”, IoT-Lite Ontology defines new concepts such as “iot-lite:Object” and “iot-lite:Service” which have become the core concepts in this model. It focuses on key concepts which support interoperability among different IoT platforms [75] by adopting lightweight semantics. [76] presents a set of information models based on IoT-Lite ontologies in which sensors are regarded as abstract components. The experiments show that the proposed models provide better data aggregation in sensors networks. According to oneM2M standard [77], an IoT-O is proposed including the definition of sensors, services, units as well as nodes, things and actuators. In addition, Ahvar [78] proposes a FUSE-IT ontology funded by Facility Using Smart secured Energy and Information Technology project, in which it combines several existing models including SAREF and SSN in order to provide a unified overview of smart homes.

(2) Ontology Editors

As ontology modeling has become a representative approach to adding semantics, more and more researchers are paying attention to developing automatic tools assisting in modeling ontologies. Among all tools, the most popular one is Protégé [79], which is developed by the Stanford Research Center based on Java language. The software is mainly used for ontologies editing in semantic web. Protégé has the compatibility of Open Knowledge Base Connectivity (OKBC) [80] in the classes and attributes, and its forums are consistent with Protégé Axiom Language (PAL) [81]. One important advantage of Protégé is the higher compatibility with different ontology description languages. WebOnto [82] is developed by Open University, UK, which is described in Options Configuration Modeling Language. Compared with Protégé, WebOnto supports multiple users to cooperate together for building ontologies. Besides, OntoEdit [83] is developed by Karlsruhe University in Germany. With the employment of graphics, it can define the mapping from concepts to vocabulary with professional view windows. It also enables many plug-in units which improve the expansibility and user-friendliness.

Moreover, there are many other automatic tools for ontology modeling such as Ontosaurus [84], WebODE [85], Ontolingua Server [86] and so forth. Therefore, selecting an appropriate tool seems much more essential. Su [87] makes a comparison of six tools including Ontolingua Server, WebOnto and Protégé in terms of Physical Quality, Empirical Quality, Semantic Quality, Syntactic Quality, Perceived Semantic Quality and Social Quality. In addition, Kapoor [88] reviews all available tools such as Protégé, Apollo, IsaViz and SWOOP in terms of interoperability, openness, and the easiness to update and maintain. All tools have their own advantages and disadvantages, and it is crucial to select the most suitable tool based on specific circumstances. A comparison between different ontology editors is presented in Table 2 from aspects of whether supporting Cooperation Work, Ontology Library, Expressivity and Consistency Check.

#### 3.3.3. Semantic Annotators

Creating semantic models is only the first step in semantic annotation, sensor data need to be instantiated according to the predefined models. Automatic semantic annotators are developed in order to improve the efficiency. Reeve [89] goes through automatic semantic annotators and makes a comparison between them in terms of Precision, Recall and F-measure. Table 3 illustrates the relationships between semantic platforms and different semantic models. With the help of the mapping relationships, users would find it easier to choose the most appropriate semantic annotator.

#### 3.3.4. Analysis and Conclusions

In this section, a comparison between different ontologies is given. Table 4 compares differences of ontologies for activity recognition in terms of Activity Granularity, Social Interoperability and Fuzzy Inference. Table 5 gives a comparison of various context ontologies from the perspective of the described classes.

In general, ontology modeling has become the mainstay of data semantization in Internet of Things. Firstly, ontology modeling replaces a great deal of labor and time in adding semantic notations, especially with the help of automatic ontology editors. Secondly, ontologies give a standard description of time, location, sensors, devices, context environments, users and activities, in a way that allows sharing and interaction between heterogenous data sources. Ontologies also have a strong reasoning ability which is significant when doing logic inference. Furthermore, ontologies have higher flexibility which enables modeling concepts in different levels. For instance, when a new sensor appears, it can be added as an instance of the abstract concept of Sensors, instead of changing the whole architecture totally. To sum up, ontology modeling has shown a greater performance in the ability of information sharing and interaction, and with the ability of logic inference, it allows intelligent agents to reason about current situation and make timely response.

## 4. Applications

Data semantization has been applied into many intelligent areas such as smart homes, e-health, smart cities and many more. With the addition of semantic notifications, it is possible to achieve deep data analysis and knowledge discovery, particularly in activity recognition, decision making and trend discovery. This section depicts typical applications of data semantization in smart homes, e-health and smart cities.

### 4.1. Smart Homes

Smart Homes are intelligent environments augmented with diverse sensors, actuators and devices. The primary goal of smart homes is to monitor inhabitants’ activities in order to enhance the quality of life and enable assisted living. Researchers all over the world are devoted to the developments of Smart Homes. However, one inevitable challenge in Smart Homes is the heterogeneity [9] which makes information difficult to communicate and interact with each other. Data semantization plays an essential role in activity recognition, risk detection and decision making in Smart Homes. Chen [5] once puts forward a new concept named Semantic Smart Home (SSH) which adds semantic notifications into sensor data. By incorporating a Semantic Layer between Data and Application Layers, it enables seamless data interoperability, integration and sharing. Entities, situations and activities can be described in unified conceptual models. It shows enormous potential in behavior monitoring and recognition based on semantic ontological models with compelling advantages such as scalability and inferability. Vlachostergiou [94] also adopts semantic technologies in Smart Homes by representing the appliances, the location, the sensor and the person with ontologies. Additionally, home rules are described in Semantic Web Rule Language (SWRL) [95] and used for activities recognition in Smart Homes. Apart from activity recognition, Huang [96] pays attention to risk recognition in Smart Homes. He proposes ontologies in terms of home context, person activity, risk and service. The semantic models focus more on risks including degrees and situations used in risk recognition in Smart Homes. Tang [97] realizes semantic decision supporting models with Semantic Decision Tables (SDT) which are annotated with domain ontologies. The SDTs model user preferences and provide proper decisions based on predefined rules.

Enormous amount of work on data semantization with regard to Smart Homes is in progress. The SESAME-S [98] is a project aimed at helping inhabitants make appropriate decisions and control energy consumption. It employs ontology-based modeling approaches to describe an energy-aware home including general concepts, data and pricing ontologies. In 2012, the SESAME system was installed in two real buildings and it did offer energy optimization in Smart Homes. The U-Health smart home project at POSTECH [99] is designed for elderly monitoring and providing appropriate support. It proposes The Smart Home Ontology Model (SHOM) which contains 103 classes and 73 relationships including health state and activity state. An automatic decision making system is built based on SHOM in order to provide better services for inhabitants in Smart Homes.

### 4.2. E-Health

The concept E-health was first proposed in 1999, and it is a health care revolution driven by electronic processes. Compared with Smart Homes, E-health data are much more complex and distributed in nature. Thus, adding semantic notifications seems more significant for interoperability and sharing between E-health data. In the agenda of European Union’s E-health policy, The European Patient Summary (EPS) infrastructure [100] is a project designed for overcoming heterogeneity of patients’ data with semantic techniques such as RDF and triplespace computing. In EPS, the patient’s data are described in ontologies which enable interaction and sharing between different types and formats. In US, the Centers for Disease Control and Prevention establishes the Public Health Information Network (PHIN) [101], in which medical data are represented in semantic models. By incorporating semantic models, PHIN makes it possible for heterogenous data exchange and information sharing.

In the area of E-health, data semantization is mostly used for providing healthcare services. Lee [102] proposes eHealth Recommendation Service System (eHeaRSS) which recommends healthcare services for patients. In this system, four static ontologies are developed including diseases, departments, symptoms and doctors, which are used for inferring diseases and generating recommendations. eHeaRSS has demonstrated a higher accuracy in supporting qualified health services compared with other DB-based health services. Vannieuwenborg [103] develops a nurse call system based on a dedicated Discrete Event Simulation (DES) model which consists of the Ambient-Aware Continuous Care Ontology [104] and rules. It is shown that the DES model is qualified to allocate patients’ calls to the most appropriate nurses.

### 4.3. Smart Cities

The Smart City has become an overwhelming trend in order to manage huge amount of pressure in water, energy and transportation brought by urbanization. However, with the lack of interoperability between heterogenous data, it is difficult to dig out deep information and knowledge. Therefore, many projects are aimed at tackling interoperability issues in Smart Cities. OpenIoT was firstly presented in 2012 which is the first open source platform to connect sensors to Cloud. It represents the Internet-connected objects by ontologies, semantic models and annotations along with semantic open-linked data techniques [105]. The CityPulse [106] proposes a framework aimed at processing data streams with semantic annotations in Smart Cities. It adopts SSN and other semantic technologies, and defines 10 application scenarios including transportation and public parking. In addition, to create advanced Smart Cities, the VITAL platform [107] enables data integration and interoperability in Smart Cities by designing VITAL ontologies including sensors, Smart Cities, IoT systems and so forth.

All of these projects are independent and isolated, and they pay little attention to compatibility issues with other projects. Thus Gyrard [108] puts forward a top level of semantic engine which provides interoperability between different projects by creating unified models, architectures and services.

## 5. Challenges and Open Issues

Data semantization is expected to provide a better understanding of heterogenous data to enable application areas such as E-health and Smart Homes become much more intelligent. With the developments and enhancements in techniques, challenges and issues are getting more attention by researchers to overcome gaps and handle them efficiently in future.

### 5.1. Standardization and Generalization

A future challenge needed to be tackled is achieving total standardization and generalization. Different applications have their own knowledge which has little interoperability with others. Even with the same observed object, granularity of the descriptions would be distinct. Organizations like OGC and W3C have proposed some industry standards such as SOS, SML, and TML [33], which are aimed at providing unified standards. By merging acknowledged standards with semantic expression formats, it is possible for heterogenous data to interact with each other.

### 5.2. Complexity and Dynamicity

Although data semantization shows great ability in representing and mining information behind data, the volatility of the environments can not be ignored. In many scenarios, when the environment changes, the semantic descriptions need to be updated. Thus, adapting semantic expressions with the changes of external environments is a tough issue to be discussed in future research. Bermudez-Edo [75] shows that the dynamicity of ambient environments is a challenge needed to be tackled in data semantization, and moreover, complexity is also an urgent issue. Kolozali [109] provides a comprehensive analysis for different segmentation methods dealing with dynamicity of environments. The results indicate that more adaptive algorithms need to be developed for better performance in dynamic sensory environments. Therefore, data semantization should take complexity and dynamicity into considerations for better application. An automatic or semiautomatic mechanism for adapting semantic notifications according to the fluctuations of environments shows great significance in providing up-to-date semantic notifications.

### 5.3. Security and Privacy

Nowadays, it is required for the techniques in data semantization to be applied into areas like smart homes and smart cities. All data collected from the sensors are personal, and represent the status of users and environments. With users paying more and more attention to security and privacy, adding privacy protection to data semantization has become an urgent requirement. Although sensors deployed in surrounding environments are better in privacy protection compared with other devices, a user defined access mechanism still holds an essential position in the process of data semantization. When sensor data are faced with semantization, the access mechanism gives controls on who can access the data, when and where can data be visited and which parts can be used. In future work, researchers need to develop security and privacy techniques equipped with data semantization.

## 6. Conclusions

Current tendency shows that data semantization in IoT has become an essential part of daily life. It provides possibilities for knowledge interaction and sharing. Ontology modeling stands out a lot in adding semantics with the standardized description formats which give great ability to merge and exchange heterogenous information. The contribution of this survey consists of a general description of data semantization in IoT, including related concepts, general architectures, key techniques, applications and challenges. Techniques involved in data semantization have been introduced, and it is true that ontology modeling has become the most pervasive technique until now. Every entity, context, user and activity can be modeled through ontologies, with strong expressivity, expansibility and reasoning ability. This paper provides a general overview of data semantization, and makes a comparison betweeen different ontology models and automatic tools. Finally, the survey analyzes challenges and open issues including the standardization and generalization, complexity and dynamicity as well as security and privacy. This is a valuable area which will show great influence on future industry.

## Figures and Tables

**Table 1:** Differences among OWL sub-languages.

Name	Inference	Type Separation	Items Restricted for Usage
OWL FULL	Undecidable	Non-mandatory	None
OWL DL	Decidable	Mandatory	RDF(s) language constructor, Role
OWL Lite	Decidable	Mandatory	RDF(s) language constructor, Role, Class constructor, Cardinality Restriction

**Table 2:** Comparison of Different Ontology Editors.

Type	Cooperation Work	Ontology Library	Expressivity	Consistency Check
Protégé	N	Y	Y	Y
WebOnto	Y	Y	Y	Y
OntoEdit	Y	Y	Y	Y
Ontolingua Server	Y	Y	N	N
Ontosaurus	Y	Y	Y	Y
WebODE	Y	N	N	Y

**Table 3:** The Mappings between Semantic Annotators and Semantic Models.

Semantic Annotators	Semantic Models
AeroDAML [90]	DAML
KIM [91]	KIMO
M3 Semantic Annotator [11]	M3
MnM [92]	Kmi
SemTag [93]	TAP

**Table 4:** Comparison of different ontologies for activity recognition.

Name	Activity Granularity	Social Interoperability	Fuzzy Inference
SOUPA	Action	N	N
CoBrA-Ont	Action	N	N
CoDAMoS	(Task, Acitivty)	N	N
PalSPOT	Activity	Y	N
The Delivery Context Ontology	N	N	N
Fuzzy-Onto	(Actions, Activities, Behaviours)	Y	Y

**Table 5:** Comparison of different ontologies for context.

Name	Service Modeling	Actuation Modeling	Electronic Labels Modeling
SSN	N	Y	N
IoT-ontology	Y	Y	Y
IoT-Lite	Y	Y	Y
IoT-O	Y	Y	N
